# Process Optimization for the Continuous Production of a Gastroretentive Dosage Form Based on Melt Foaming

**DOI:** 10.1208/s12249-021-02066-y

**Published:** 2021-06-21

**Authors:** Ádám Haimhoffer, Gábor Vasvári, György Trencsényi, Monika Béresová, István Budai, Zsuzsa Czomba, Ágnes Rusznyák, Judit Váradi, Ildikó Bácskay, Zoltán Ujhelyi, Pálma Fehér, Miklós Vecsernyés, Ferenc Fenyvesi

**Affiliations:** 1 Department of Pharmaceutical Technology, University of Debrecen, Nagyerdei krt. 98, Debrecen, H-4032 Hungary; 2 Doctoral School of Pharmaceutical Sciences, University of Debrecen, Nagyerdei St. 98, Debrecen, H-4032 Hungary; 3 Institute of Healthcare Industry, University of Debrecen, Nagyerdei St. 98, Debrecen, H-4032 Hungary; 4 Department of Medical Imaging, University of Debrecen, Nagyerdei krt. 94, Debrecen, H-4032 Hungary; 5 Faculty of Engineering, University of Debrecen, Ótemető utca 2-4, Debrecen, H-4028 Hungary

**Keywords:** gastroretentive, floating formulation, melt foaming, continuous production

## Abstract

Several drugs have poor oral bioavailability due to low or incomplete absorption which is affected by various effects as pH, motility of GI, and enzyme activity. The gastroretentive drug delivery systems are able to deal with these problems by prolonging the gastric residence time, while increasing the therapeutic efficacy of drugs. Previously, we developed a novel technology to foam hot and molten dispersions on atmospheric pressure by a batch-type in-house apparatus. Our aim was to upgrade this technology by a new continuous lab-scale apparatus and confirm that our formulations are gastroretentive. At first, we designed and built the apparatus and continuous production was optimized using a Box–Behnken experimental design. Then, we formulated barium sulfate-loaded samples with the optimal production parameters, which was suitable for *in vivo* imaging analysis. *In vitro* study proved the low density, namely 507 mg/cm^3^, and the microCT record showed high porosity with 40 μm average size of bubbles in the molten suspension. The BaSO_4_-loaded samples showed hard structure at room temperature and during the wetting test, the complete wetting was detected after 120 min. During the *in vivo* study, the X-ray taken showed the retention of the formulation in the rat stomach after 2 h. We can conclude that with our device low-density floating formulations were prepared with prolonged gastric residence time. This study provides a promising platform for marketed active ingredients with low bioavailability.

## INTRODUCTION

Oral drug delivery systems are the most common and convenient forms to administer drugs due to their various advantages such as good compliance, low costs for storage and transport, and various forms which can be manufactured [1]. On the other hand, the development of a formulation of oral drug delivery systems is highly challenging due to the physiological variability of the gastrointestinal tract. The pH varies in different parts of the digestive tract, and the motility depends on meals, not to mention the enzyme activity [2, 3]. Several drugs have poor oral bioavailability due to low or incomplete absorption which is affected by the various effects as mentioned above [4]. In addition, some of the active ingredients (API) have an absorption window that is located on the upper part of the gastrointestinal tract: these are the stomach, jejunum, and duodenum in many cases [5]. Conventional drug delivery systems may not overcome the special environment of the digestive system. The gastroretentive drug delivery systems are able to deal with these problems, by prolonging the gastric residence time while increasing the therapeutic efficacy of drugs [6]. To succeed in gastric retention, several technologies are available nowadays [2, 4]. Mucoadhesive formulations contain adhesive biopolymers that adhere to the mucosa covering the stomach inner wall and release their API in a sustained manner [7–10]. Expanding devices inhibit transit through the pyloric sphincter, due to size increase by getting in contact with gastric juice [11]. There are countless possibilities to develop low-density drug carriers [12–14]. The first formulation was published by Sheth and Tossounian in 1975 [15]. The most important feature of these systems is that their density is below 1.00 g/cm^3^, and thereby, they float on the surface of the gastric fluid. Another major grouping principle is the mechanism of gas formation: gas-generating [16] and non-gas-generating systems [13, 17]. Gas-generating formulations usually contain carbonates and a polymer mixture to entrap the formed gas after the administration [18]. To raise these formulations to the surface, a couple of minutes is necessary; however, the immediately floatable devices, the non-gas-generating systems, have no lag time and they remain on the liquid surface [19]. To date, the promising new technologies to produce low-density gastroretentive drug delivery systems are hot melt extrusion and melt foaming [13, 20]. Previously, we developed a novel technology to foam hot and molten dispersions on atmospheric pressure. This technology is directly applicable to produce immediately floating, low-density molded solid dosage forms by a batch-type in-house apparatus. Some of our products that contained SA and PEG 4000 mixture reached a density lower than 1 g/cm^3^, which made them suitable for immediate floating in acidic buffer without the need of gas generation and entrapment [20]. After foaming, the density test and *in vitro* drug release test proved the ability of gastroretention; thus, it raised the issue and implementation of industrial manufacturability [20].

Our aim was to upgrade this technology that was based on melt foaming. In this study, the prototype of a novel apparatus is presented, which allows the continuous lab-scale production and is suitable to fill the foam into hard gel capsules. Beyond the determination of the key parameters, we confirmed that our formulations are gastroretentive, proven by *in vitro* and *in vivo* studies. We applied BaSO_4_ as an API to prepare and test the foams suitable for computer tomography (CT) imaging.

## MATERIALS AND METHODS

### Materials

Polyethylene glycol 4000 (PEG4000), stearic acid, type 50 (SA), lactose monohydrate, and barium sulfate (BaSO_4_) were Ph. Eur. grade and purchased from Molar Chemicals Ltd. (Halásztelek, Hungary). Other reagents were analytical grade and purchased from Sigma-Aldrich Ltd. (Budapest, Hungary). The hard gelatin capsules (Coni-Snap, size 00) were gifted by Capsugel (Morristown, NJ, USA). Fischer-344 rats were bred by Animalab Ltd. (Budapest, Hungary).

### Methods

#### Development of the Foam Cell Device

**Fig. 1:**
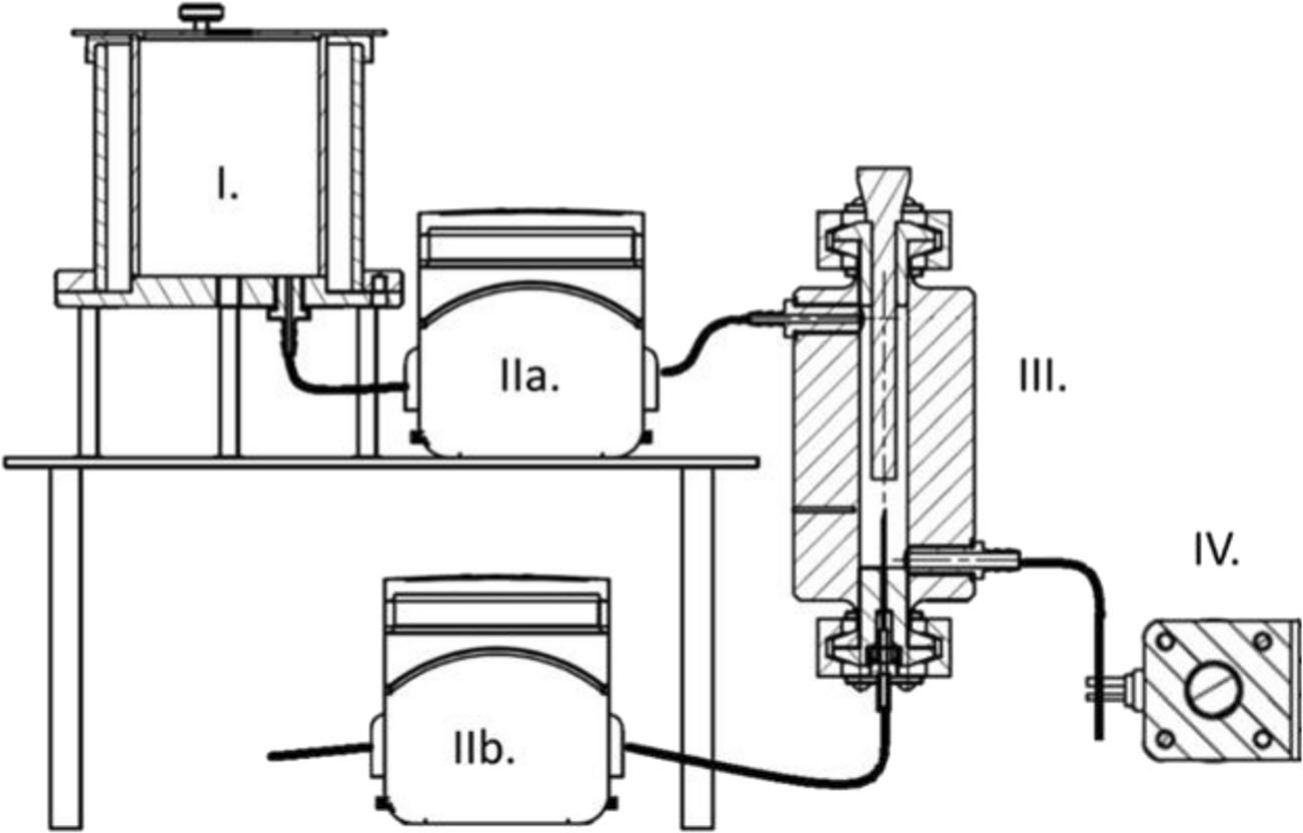
The main parts of QUICKfoamcell. The figure of the equipment is not to scale only for better comprehensibility. (I.) Melt container. (IIa.) Inlet pump for dispersion. (IIb.) Inlet pump for gas. (III.) Foam cell. (IV.) Pinch valve

The QUICKfoamcell Lab® was designed in cooperation with QUICK 2000 Ltd. (Tiszavasvári, Hungary) and built from stainless steel. The equipment is presented in Fig. 1. The apparatus can be divided into two main parts: a vessel with the volume of 600 mL (melt container), which has 8-mm-wide drainpipe at the bottom; IKA EURO-ST D overhead stirrer with 4-bladed propeller stirrer protruding into the container continuously mixing the melt. The drainpipe is connected to a Watson-Marlow 114 ST peristaltic pump which transfers the homogeneous melt into foam cell. The foam cell capacity is 30 mL with 3 openings: inlet for the dispersion, gas inlet, and outlet for foamed product. The agitation is done by IKA® ULTRA-TURRAX® T-25 Digital disperser equipped with a dispersing tool (S25 N - 10G). Gas is introduced to the melt by another Watson-Marlow 114 ST peristaltic pump. Dosing is controlled by TAKASAGO PK–6405–NC pinch valve. The melt container and foam cell are tempered by mantle heating.

#### Manufacturing of Foam

**Fig. 2:**
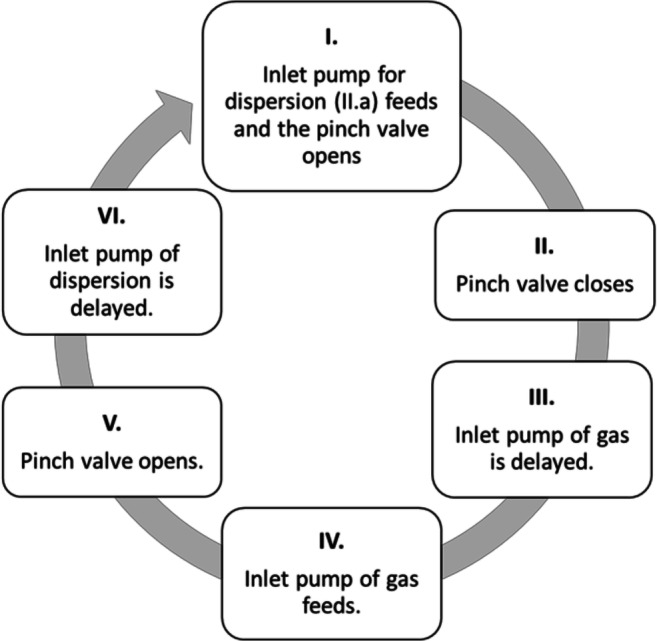
Steps of continuous foaming. Firstly, the inlet pump of dispersion feeds 2 mL dispersion into the foam cell with 0.25 mL/s, while the pinch valve opens to dose the foam into the 00 capsule. Then, the pinch valve closes and the inlet pump adds gas with a specified volume to produce foam after a 1000-ms delay. Later, the pinch valve opens and after 4000 ms the inlet pump of dispersion starts the dosing

A total of 150 g of melt was foamed by the following method. PEG 4000 and SA were measured and melted in a melt container while gentle stirring was used with 50 rpm by IKA EURO-ST D overhead stirrer. Then, lactose or BaSO_4_ was dispersed in the melted mixture. Firstly, the apparatus was filled with the molten suspension by a peristaltic pump (IIa.). The continuous foaming was carried out in cycles. One cycle consists of 6 steps, presented in Fig. 2. From steps 1 to 6, the process went cyclically while having high agitation by IKA® ULTRA-TURRAX® T-25 Digital disperser. After every cycle, the hot foam was filled into 00 size hard gel capsules and allowed to cool to room temperature.

#### Effect of Temperature on Foam Production

The rate and extent of the successful foaming are strongly affected by the temperature used in the process, as described in our previous publication [20]. To determine the effect of temperature on the density of foams, 20 capsules were produced at different temperatures, while the other parameters were left unchanged. The temperature of the foam cell was set to 56, 58, or 60°C; the applied volume of gas was 2 mL with 0.25 mL/s gas injection rate. The agitator shaft was kept at 15,000 rpm speed. Control samples were prepared without the foaming process and the samples contained only the matrix of foams without lactose.

The density of the produced foams was determined by the following method. The weight and the volume of the 00 size capsule were 118 ± 7 mg and 0.91 mL according to the manufacturer’s specification [21]. Every capsule was completely filled with melt and after the solidification, the capsules were sealed with cap. The filling weight contained exactly 0.91 mL solid foam and was measured by an analytical balance, and the density of each sample was determined with the following formula:

$$ {\rho}_{foam}=\frac{m_{sample}-{m}_{capsule}}{V_{capsule}} $$

where V_capsule_ is the volume of the empty capsule (0.91 mL); m_sample_ is the weight of sample with the capsule body and cap; m_capsule_ is the weight of the empty capsule with cap (118 mg); ρ_foam_ is the density of the prepared foam.

#### Optimization of Process Parameters

The continuous production was optimized using a Box–Behnken experimental design [22]. The independent variables were the volume of gas (mL), gas injection rate (mL/s), and agitator shaft speed (rpm), and considered the critical parameters in the production process with an effect on product density. These three experimental factors are varied in the design, at 3 levels in 12 runs, presented in Table I. The volume of gas was changed between 0.1 and 4 mL, while the gas injection rate was from 0.02 to 0.5 mL/s and the speed of the agitator was set from 3000 to 25,000 rpm. This design was employed to investigate the quadratic response surface and to construct a second-order polynomial model using TIBCO Statistica® 13.4 (StatSoft Hungary, Budapest, Hungary).

**Table I:** Box–Behnken Experimental Design of Foam Production

Standard run	Speed of agitator (rpm)	Volume of gas (mL)	Gas injection rate (mL/s)
1	3000	0.1	0.02
2	3000	2	0.5
3	3000	4	0.25
4	14,000	0.1	0.5
5	14,000	2	0.25
6	14,000	4	0.02
7	25,000	0.1	0.25
8	25,000	2	0.02
9	25,000	4	0.5
10	14,000	2	0.25
11	14,000	2	0.25
12	14,000	2	0.25

The 3D response surface plots for density were plotted according to the regression model by keeping one variable at the center level. For statistical analysis, GraphPad Prism® (Version 6.01, GraphPad Software Inc.) was used. Unpaired t-tests were performed when two groups were compared, and one-way ANOVA was chosen when comparison of multiple groups was performed. Differences were considered significant at *p* < 0.05.

#### Preparation of Floating BaSO_4_-Loaded Samples

The barium sulfate samples, containing 30% BaSO_4_, 50% PEG 4000, and 20% stearic acid, were foamed based on the method mentioned above. The independent variables were set to 2.5 mL of gas injected with 0.25 mL/s rate at 15,000 rpm agitator shaft speed. The foam cell was set to 54°C. The hot foam filled the plastic tubes (d: 5 mm, h: 50 mm). After cooling to room temperature, the rods were cut to uniform size (d: 5 mm, h: 5 mm).

#### Determination of the Density of BaSO_4_-Loaded Samples

The following method was used to determine the density of the solid compositions (unfoamed or foamed) [20]. The shape of BaSO_4_-loaded samples is cylindrical with a diameter of 5 mm and a height of 5 mm and the top and bottom are perpendicular to the mantle; thus, the method of calculating the volume used for the cylinder is usable. The volume of the samples was calculated from the dimensions of the cylinder:

$$ {V}_{sample}={\left(\frac{d}{2}\right)}^2\times \pi \times h $$

where V_sample_ is the volume of the cylinder-shaped sample; d is the diameter of the samples (5 mm); π is the mathematical constant; h is the height of the sample.

The total weight was checked by an analytical balance and the density of each sample was determined with the following formula:

$$ {\rho}_{foam}=\frac{m_{sample}}{V_{sample}} $$

where m_sample_ is the weight of the BaSO_4_-loaded sample; V_sample_ is the volume of the cylinder-shaped final preparation (0.0981 mL); ρ_foam_ is the density of the prepared foam.

#### Microtomography and Size Distribution of Foam Bubbles

The following method was used to determine the solid foam structure. The tablet was fixed into the sample holder. A SkyScan 1272 compact desktop microCT system was used for the measurement. Scanning parameters were the following: image pixel size, 5 μm; matrix size, 1344×2016 (rows × columns); source voltage, 50 kV; source current, 200 μA; flat field correction and geometrical correction were used. After scanning, SkyScan NRecon package (Version: 2.0.4.2) was used to reconstruct cross-section images from tomography projection images. Post-alignment, beam-hardening correction, ring artifact correction, and smoothing were done. The output formats were DICOM and BPM images.

In 2D/3D analysis, we used CTAn software. Based on density analysis, we used Thresholding, ROI shrink-wrap, Reload, and 2D and 3D Analysis plugins. The gray threshold values of air bubbles were between 0 and 40, and with ROI shrink-wrap we eliminated the background before analysis. The 3D visualization can be seen in CTVox software with color coding.

#### Texture Analysis

The mechanical properties and structure of the dry and wetted foamed compositions were characterized by texture analysis. Dry samples were tested at room temperature without immersing them in dissolution media. To monitor the erosion and changes in hardness of BaSO_4_**-**loaded samples, three random samples were placed into beakers containing 50 mL of pH 1.2 hydrochloric acid media and stirred at 37°C (Velp AREX-6 Digital heating magnetic stirrer, 100 rpm). The samples were carefully removed 0.5, 1, 2, and 4 h later and excess water was carefully removed by soft tissues from the samples. Wet and dry samples were analyzed by Brookfield CT3 texture analyzer. An acrylic cylinder, TA25/1000 (d: 50.8 mm), compressed the samples with constant speed (0.50 mm/s) until 4500 g of load. At the target pressure, the device fixed the probe for 5 s as the hold time. Following the hold time, the probe returned to its initial position. The load (g) values were plotted in the function of time (s) to present the changes in the texture in real time.

#### *In Vivo* Gastroretentive Study

*In vivo* gastroretentive study was performed to confirm the retention of the BaSO_4_-loaded samples in the stomach, for the suitability of the technology for drug development. BaSO_4_ is a contrast agent that is used in clinical imaging area with high-density and high X-ray absorbing properties which make it suitable to determine the intracorporeal location of the compositions [23, 24].

##### Experimental Animals

For the *in vivo* experiments, 16-week-old, 250–300-g weighted male Fischer-344 rats (n=3; Animalab Ltd., Budapest, Hungary) were used. Animals were housed under conventional conditions at 23±2°C with 50±10% humidity and artificial lighting with a circadian cycle of 12 h. The semi-synthetic diet (VRF1; Akronom Ltd., Budapest, Hungary) and drinking water were available *ad libitum* to all animals. The animal experiments did not apply invasive techniques; ethical permission was not required to do the investigations. The laboratory animals were kept and treated in compliance with all applicable sections of the Hungarian Laws and animal welfare directions and regulations of the European Union.

##### *In Vivo* CT Imaging

Rats were anesthetized by 3% isoflurane (Forane) with a dedicated small animal anesthesia device, and barium containing minitablet was administrated per os directly into the stomach of the animals [25]. For the anatomical localization of the tablet containing 15 mg BaSO_4_/tablet, prepared by the method described in section “Preparation of Floating BaSO4-Loaded Samples,” whole-body CT scans were acquired after 0.5 and 2 h using the nanoScan SPECT/CT (Mediso Ltd., Hungary) scanner. The following acquisition parameters were used: X-ray tube voltage 60 kVp, current 86 mA, exposure time 170 ms per projection, voxel size 1×1 mm.

## RESULTS

### Optimization of Process Parameters

**Fig. 3:**
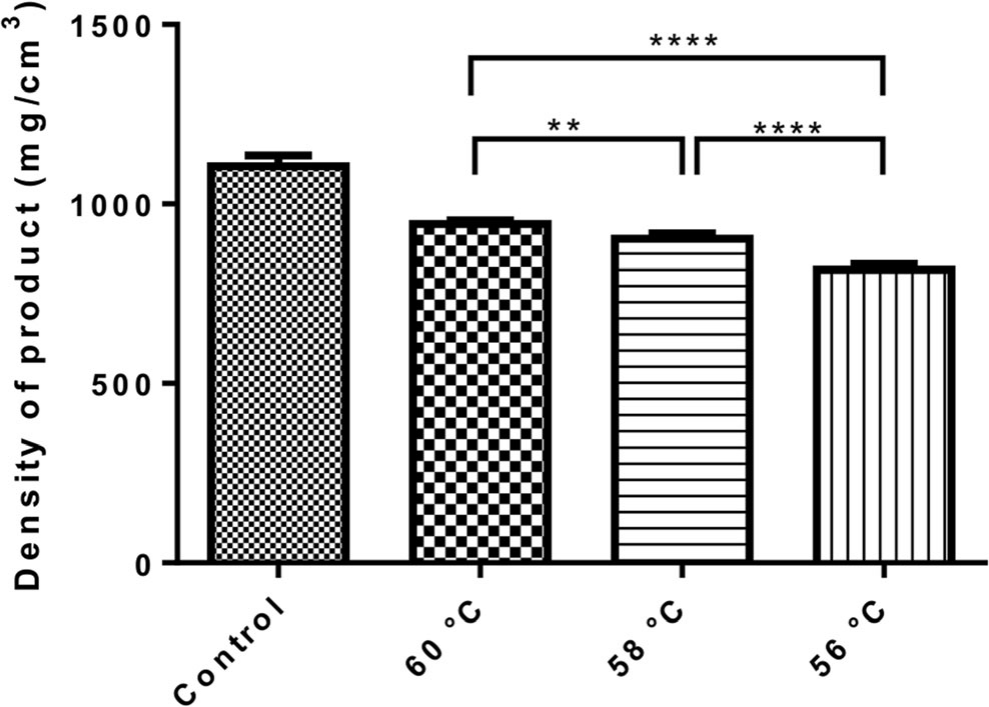
The effect of foaming temperature on the density of the foamed samples. All of the foamed samples showed significantly lower densities compared to the unfoamed control (*p*<0.0005). In comparison, there is a statistically significant difference in the density of products produced at different temperatures. ** and **** indicate statistically significant differences at *p* < 0.01 and *p* < 0.0001. Data present average values and standard deviations (n = 5)

The relationship of viscosity and gas entrapment efficacy was described in our previous publication. It was revealed that during the precise cooling, the viscosity values increased as the molten dispersion became semi-solid from its liquid state [20]. The freezing range of molten mixture of PEG4000 and SA was around 53–55°C. The production temperature was a key parameter to maximize gas entrapment efficacy additionally avoiding the freezing during the foaming process. The effect of the temperature was determined by manufacturing at 60, 58, and 56°C; the results are presented in Fig. 3. The temperature 56°C was found to possess the lowest density, namely 817.14 mg/cm^3^.

**Fig. 4:**
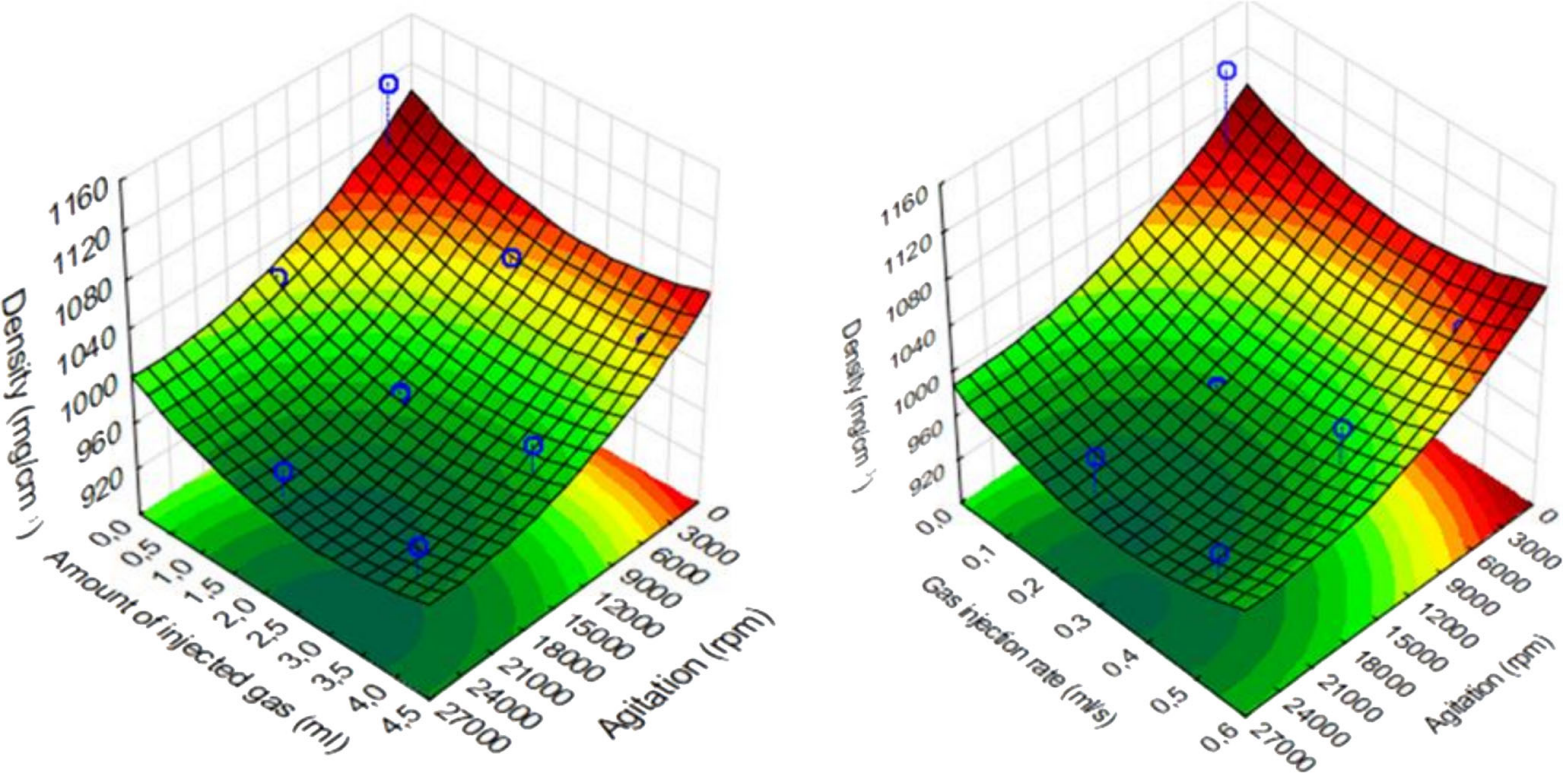
Three-dimensional illustration of the density changes during the three-factorial experimental design

The effect of the volume of gas (mL), gas injection rate (mL/s), and agitator shaft speed (rpm) on density of samples was also investigated. In this study, the temperature was set at 56°C. The results are presented in Fig. 4. Increasing the speed of agitator shaft decreased the density of samples in every case. To reach optimal density (<1000 mg/cm^3^) with all settings, 14,500 rpm was enough. In the case of volume of gas and speed of gas, it was found that the degree of foaming decreases at the extreme values. As a result of rapid introduction of higher levels of gas, the rate of foaming is reduced. On the other hand, at lower ranges of gas injection rate or at low levels of gas, the rate of foaming decreases. The optimal parameters were the following: volume of gas, 2.5–3.25 mL; the injected speed of gas, 0.25–0.35 mL/s; and the speed of agitator 14,500 rpm. In every case to reach target density range, 14,500 rpm was enough which is lower than 1000 mg/cm^3^.

### Density of BaSO_4_-Loaded Samples

The density values of the dispersions before and after foaming were significantly different. It was found that the composition reaches 507 ± 45.48 mg/cm^3^ after the foaming process. The initial density was 1107 ± 108.18 mg/cm^3^. This means a 54% decrease in the mass due to the dispersed gas. The foamed compositions showed zero floating lag time with continuous floating until complete disintegration (over 4 h) in pH 1.2 buffer.

### Microtomography and Size Distribution of Bubbles

**Fig. 5:**
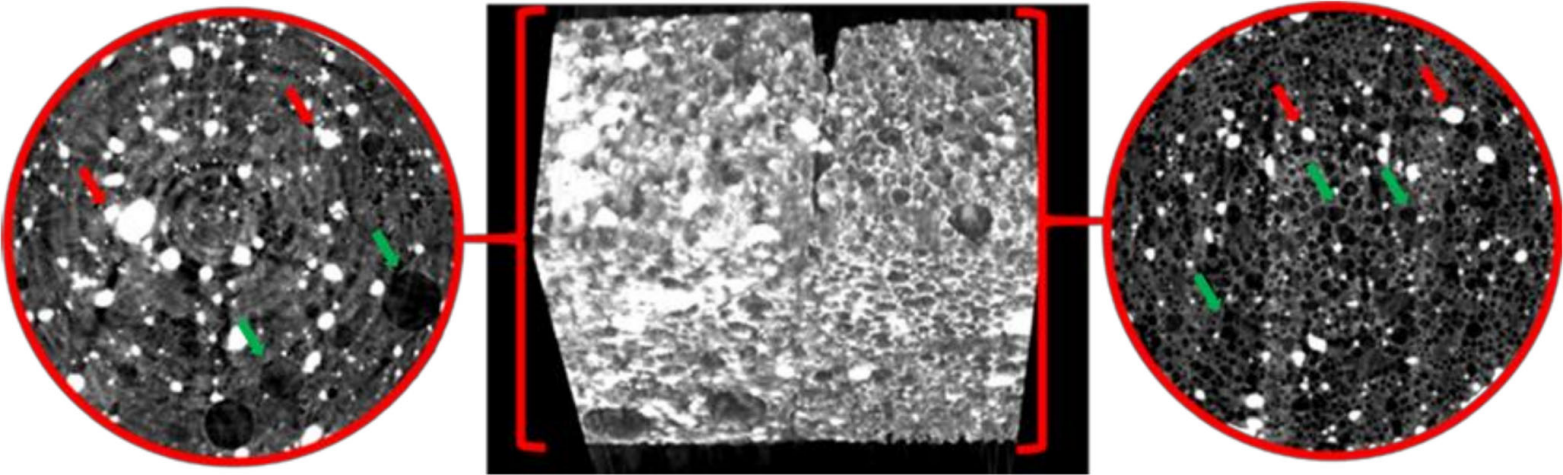
Reconstructed microCT images of the BaSO_4_ samples. The left part represents the unfoamed initial form with a section from the original images parallel to the base, while the right part represents the foamed low-density product with a section from the original images parallel to the base. The green arrows indicate bubbles, while red arrows indicate BaSO_4_ particles

**Fig. 6:**
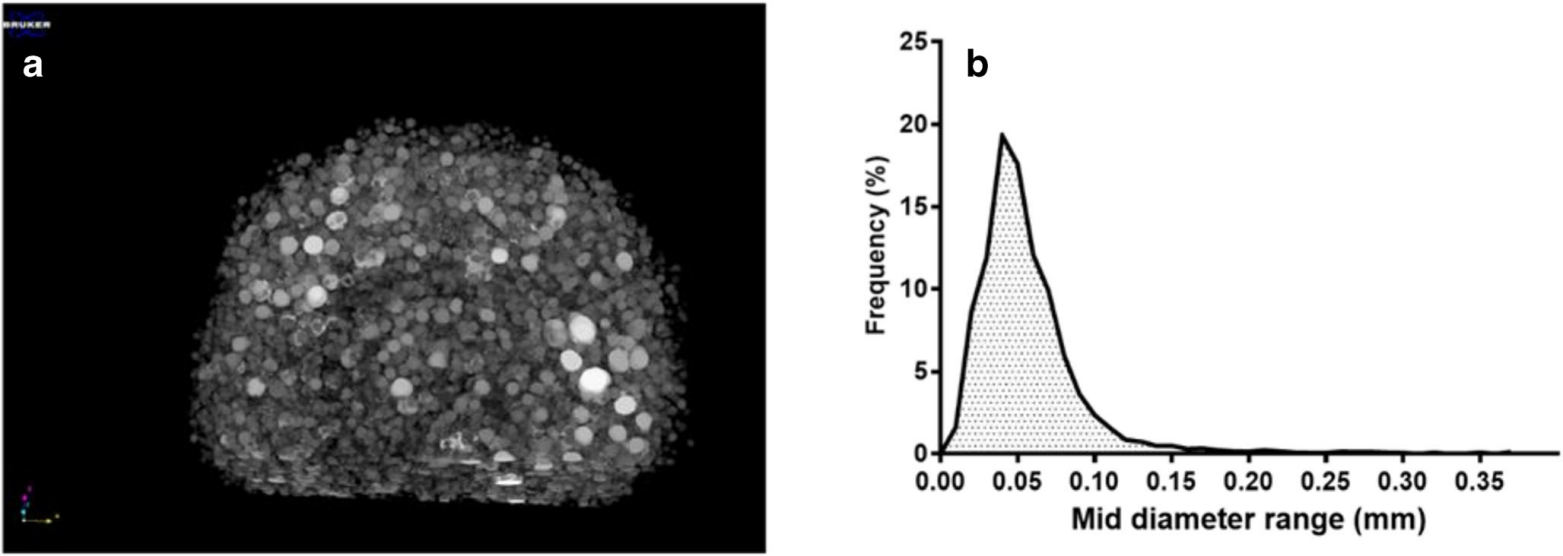
**a** Reconstructed model of spheroid closed-cell structure of the BaSO_4_ foamed sample. **b** The size distribution curve of spheroid cells

The image of microCT scans performed on BaSO_4_**-**loaded composition is presented in Fig. 5. The foaming process dispersed gas bubbles into the molten suspension which was confirmed by microCT images. On the other hand, the unfoamed composition also showed bubbles, but the number was negligible and the size was significantly higher than the foam preparation. The distribution of the bubbles was random. The reconstructed and computed model of the foam structure (Fig. 6(a)) shows a closed spheroid cell structure. The size distribution was homogenous, 93.2% of the bubbles are in the 0–100 μm range of diameter, and the average size of the bubble was around 40 μm (Fig. 6(b)).

### Texture Analysis

**Fig. 7:**
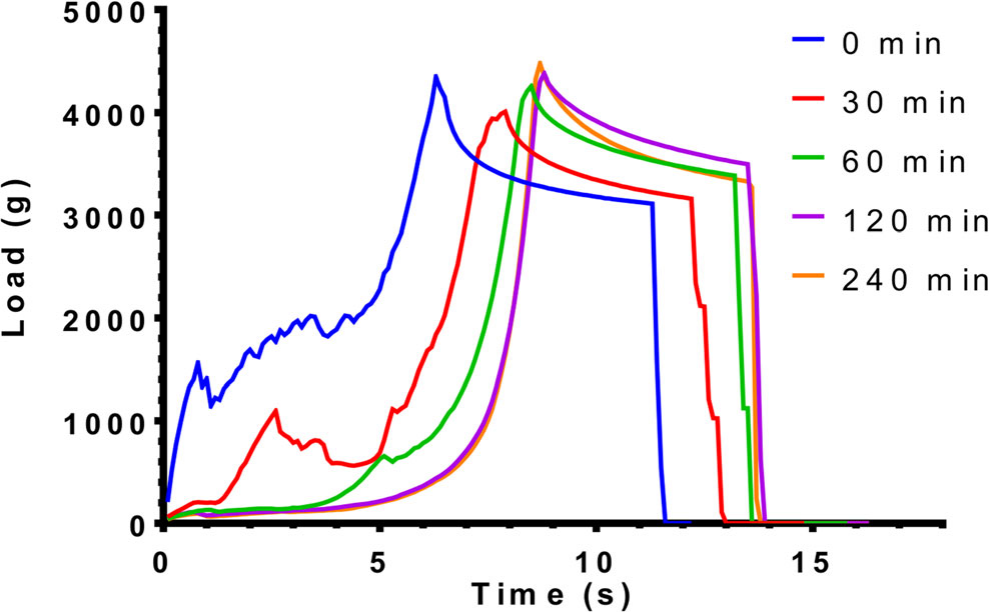
Texture analysis results of the dry, foamed composition at 25°C (0 min) and wetting coupled texture analysis result of the immersed samples at 37°C after 0.5 h, 1 h, 2 h, and 4 h. X-axis shows the time of the movement of the compression test probe

The results of the texture analysis are presented in Fig. 7. It was revealed that a hard structure is presented in spite of the air entrapment, but applying 4500 g of compression load on the foam results in cracks in the dry state at 25°C. During the erosion test, the hardness of matrix decreased and the cracks disappeared from the texture curve at the same time. Wetting caused the gelling of the outer layer of the samples; thus, the compression test probe reached the solid-resistant core later during the measurement. The complete wetting was detected after 120 min. We did not experience significant changes between the 120 and 240 min.

### *In Vivo* Gastroretentive Study

**Fig. 8:**
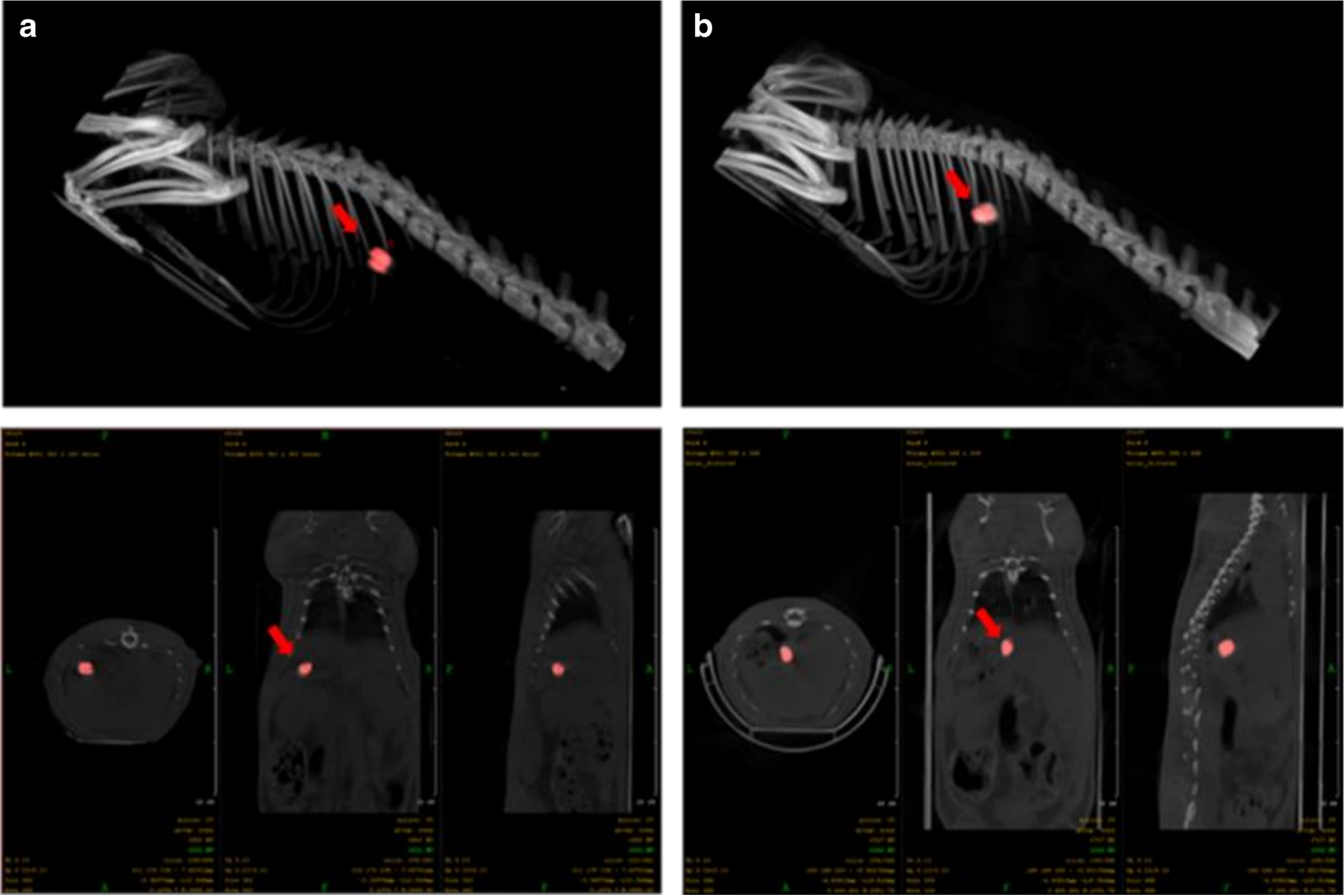
Abdominal CT images of BaSO_4_-containing gastroretentive sample in a rat. Abdominal CT images indicating the positions of the floating system with red painting and arrow, in the gastrointestinal tract of a rat after 30 min (**a**) and 2 h (**b**)

BaSO_4_ is a contrast agent that is used in clinical imaging area with high-density and high X-ray absorbing properties which make it suitable to determine the intracorporeal location of the compositions [23, 24]. Figure 8 (a) shows that the formulation appeared in a well-defined way in the stomach 30 min after the administration. As seen in the X-ray taken after 2 h, in Fig. 8(b), the retention of the formulation was confirmed in the stomach, in spite of the erosion. This study proved the ability of the foam to remain in the stomach for a prolonged period of time to satisfy the desired needs of such formulations.

## DISCUSSION

Our aim was to design, build, and optimize a novel foaming apparatus that is suitable for continuous production of foamed molten dispersions. Our device is suitable for continuous production with an approximate capacity up to 300–500 capsules/h. The QUICKfoamcell can be divided into two basic units. The melt container had a size of 600 mL, but the capacity can be easily multiplied as needed. The vessel can be heated to 70°C, which makes it suitable for various moldable polymers and materials [26]. The main unit of foaming is the foam cell, where gas is dispersed in the molten dispersion with a high agitation speed. The final dosage form is a hard capsule since the capsule shell is used as a mold in which the foamed composition can solidify. The 00 size capsule has a volume of 0.91 mL [21]. Depending on the density of the product, it can contain up to 300 mg of active ingredient, which broadly covers the therapeutic dose of most API whose bioavailability can be enhanced by gastroretention [1, 27]. During optimization of process parameters, the ideal production temperature was determined between 54 and 56°C, but the optimal temperature depends on the composition. The API might change the solidification temperature and viscosity of the melt, which affects the production temperature [20]. We found that increasing the speed of agitation causes significantly higher gas dispersion efficiency [20, 28], while the volume of the introduced gas and the gas injection rate have an optimal range. We hypothesize that a bubble plug formed around the dispersing tool at a higher range of parameters, due to larger detached bubbles. Ji Ma *et al.* published that the higher gas injection velocity increases the bubble detaching volume with a non-linear relationship [29]. The formation of bigger bubbles may cause a gas plug around the agitator causing decreased gas dispersion efficiency, which was reflected on the three-dimensional illustration of the three-factorial experimental design. The density change followed also a non-linear decrease in the function of gas injection velocity. At lower values of parameters, the foaming was also unsatisfactory, and the density of the preparation did not fall below 1 g/cm^3^. BaSO_4_ increased the melt viscosity and thus increased foaming by higher gas entrapment with a density decrease of almost 54%, while in the case of our previously reported batch production, the density of this formulation was reduced only by 36% [20]. Due to the density of 507 mg/cm^3^, the composition shows zero floating lag time with continuous floating without gas generation [19]. On the other hand, compared to other molten gas technologies, like hot melt extrusion with CO_2_, our achieved density was higher [30]. The microCT image clearly shows the white BaSO_4_ particles [31] which have a high X-ray absorption [32]. In other published studies, highly porous formulations could be achieved by forming 200–300-μm cavities [17, 20, 33]. The bubbles in the present composition show homogeneous size distribution with the average diameter of 40 μm, measured by microCT. Pores make up 44% of the composition’s volume, which is formed by bubbles, so that a very high surface area is associated with the total bubble volume. According to the achieved small bubble size, the high total volume of incorporated gas, and the homogenous distribution of pores, our composition can be considered to have a high porosity. Gas bubbles are not opened to the outer environment creating a closed-cell structure in the whole matrix. BaSO_4_ formulation is hard at room temperature but can be crushed with a compressive force of 15 N; this force is lower than the average friability of tablet or pellet with similar sizes [12], but it is strong enough to resist the grinding motions of the stomach, until complete wetting. Texture analysis showed that the complete wetting of the samples occurs in 120 min. BaSO_4_ samples remained in one piece, longer than we expected. Since BaSO_4_ has negligible solubility in aqueous media, its dissolution does not propagate matrix erosion and may contribute to preserving the hardness or texture of the sample. During compression of the sample in the texture analysis, a small amount of water was pressed out from the samples immersed in the acidic media for 4 h. It can be explained by the poor wetting angle of BaSO_4_ [34], as well. Incorporation of BaSO_4_ into test formulations is often used to check gastric retention *in vivo* [35]*.* Samples were detected and identified easily in the *in vivo* test. In light of the study, we confirmed that the BaSO_4_-containing samples own at least a 2-h-long gastric residence time. The preparations were not emptied from the stomach despite that we have noticed a slight decrease in sample sizes. Due to its low density, the current foamed dosage form is suitable for gastroretention, which has been demonstrated *in vitro* and *in vivo*, as well.

## CONCLUSION

Novel apparatus was designed and built for continuous foaming of molten dispersions. The foaming process was optimized by a Box–Behnken experimental design to determine the most effective setup to create solid foams. We developed high-porosity BaSO_4_ samples with low density, namely 507 mg/cm^3^. Texture analysis was done to characterize the *in vitro* behavior of the samples in acidic media. To test the gastric retention of the foamed formulation, insoluble BaSO_4_ was used as a contrast agent for *in vivo* imaging in a rat model. We can conclude that with our device low-density floating formulations were prepared successfully with a prolonged gastric residence time. This study provides a promising platform for marketed active ingredients with low bioavailability.
